# Digital adoption by enterprises in Malaysian industrial sectors during COVID-19 pandemic: A data article

**DOI:** 10.1016/j.dib.2021.107197

**Published:** 2021-06-04

**Authors:** Suriyani Muhamad, Suhal Kusairi, Mustafa Man, Nur Fatma Hasni Majid, Wan Zulkifli Wan Kassim

**Affiliations:** a Faculty of Business, Economics and Social Development, Universiti Malaysia Terengganu, Terengganu, Malaysia; b Faculty of Ocean Engineering Technology and Informatics, Universiti Malaysia Terengganu, Terengganu, Malaysia; c Centre for Fundamental and Continuing Education, Universiti Malaysia Terengganu, Terengganu, Malaysia

**Keywords:** Industrial sector, COVID-19, Digital tools, Enterprises

## Abstract

This article presents data on digital adoption by enterprises in Malaysian industrial sectors during the COVID-19 pandemic. The data were collected during the periods of Conditional Movement Control Order (CMCO) and Recovery Movement Control Order (RMCO) from October 11 to December 31, 2020. Data collection was completed through an online questionnaire survey conducted among a sample of 432 enterprises from four industrial sectors, namely services, retail, manufacturing, and tourism, in all states in Malaysia. The sample was selected using cluster and systematic random sampling. The questionnaire asked respondents to state whether they used the Internet, computers, phones, web sites, e-payment, and e-commerce to complete their activities relating to finance, production and operations, human resource management, and marketing. The data were analysed using descriptive statistics and cross-tabulation. The data show the extent of digital adoption by Malaysian enterprises during the pandemic in comparison to the situation before the pandemic. The data may be of use to other similar researchers as comparison and to policy makers as guides in devising related policies.

## Specifications Table

**Table:** 

Subject	Economics
Specific subject area	Economic Development
Type of data	Table Raw data (.xls) Questionnaire survey Descriptive statistics (Table 1) Cross tabulation (Table 2, Table 3, Table 4, Table 5)
How data were acquired	Online survey using the SurveyMonkey platform
Data format	Raw, processed, descriptive
Parameters for data collection	Cluster and systematic random sampling were used to select 432 enterprises from the services, retail, manufacturing, and tourism sectors as sample, who then participated in an online questionnaire survey.
Description of data collection	Data were collected during the periods of Conditional Movement Control Order (CMCO) and Recovery Movement Control Order (RMCO) between October 11 and December 31, 2020, in Malaysia. The respondents participated in an online survey through the SurveyMonkey platform.
Data source location	All states in Malaysia: Terengganu, Kelantan, Pahang, Kedah, Perak, Perlis, Selangor, Wilayah Persekutuan Kuala Lumpur, Melaka, Negeri Sembilan, Pulau Pinang, Johor, Sabah, and Sarawak.
Data accessibility	Dataset is available on Mendeley Data Repository: https://data.mendeley.com/datasets/xrh8ssskjb/1

## Value of the Data

• The data help identify the digital adoption level among enterprises in Malaysian industrial sectors before the COVID-19 and during the COVID-19 pandemic.
• The data may help other researchers compare other similar data on digital adoption by enterprises during the COVID-19 pandemic and contribute to future meta-analysis.
• The data may provide insights to policymakers when proposing policies for improving digital adoption among enterprises and promoting the importance of digital platforms, particularly during the COVID-19 pandemic.

## 1 Data Description

Due to the COVID-19 pandemic that began to hit Malaysia in early 2020, it has been challenging for enterprises to operate efficiently and safely. One of the ways that can help them maintain efficient and safe operations during the pandemic is to adopt digitalisation. It is interesting to find out to what extent this practice has existed. Presented here are data on digital adoption by enterprises in Malaysian industrial sectors during the COVID-19 pandemic.

The dataset, available on the Mendeley Data repository (https://data.mendeley.com/datasets/xrh8ssskjb/1), has two parts. The first part contains enterprises’ names, their addresses, and their sector types. The second part contains data on enterprises’ digital tool adoption according to functions and the variables used to identify their responses. Digital tools included are internet usage, computer/phone usage, web presence, e-payment, and e-commerce. Functions in which digital tools are used are finance activities (payment transactions, working capital management, financing management), production and operations activities (material handling, product design, production, quality control), human resource management activities (teleworking options for staff, evaluation of staff performance), and marketing activities (sales of goods or services, advertising). The responses covered three periods: before COVID-19; during COVID-19, specifically during the Movement Control Order (MCO) from March 18 until May 12, 2020; and the new norm phase from May 13 until December 31, 2020.

Table 1 presents the frequency distribution of respondents based on sector types. The sector type with the highest enterprise involvement was services (38.4%) and the type with the lowest was tourism (10.6%).

**Table 1:** Respondents’ sector types.

Type	Frequency	Percentage
Services	166	38.4
Retail	157	36.3
Manufacturing	63	14.6
Tourism	46	10.6
Total	432	100.0

Tables 2–5 report the frequency of digital tool adoption in finance activities, production and operation activities, human resource management activities, and marketing activities by enterprises in the services, retail, manufacturing, and tourism sectors.

**Table 2:** Digital tool adoption in services sector.

	Before COVID-19		During COVID-19 (MCO)		New norm	
Digital tool	Frequency	Percentage	Frequency	Percentage	Frequency	Percentage
**Finance**						
Internet usage	141	84.9	149	89.8	150	90.4
Computer/Phone usage	134	80.7	141	84.9	145	87.3
Web presence	37	22.3	44	26.5	50	30.1
E-payment	94	56.6	101	60.8	109	65.7
E-commerce	51	30.7	55	33.1	57	34.3
**Production and operations**						
Internet usage	96	57.8	101	60.8	103	62.0
Computer/Phone usage	92	55.4	96	57.8	100	60.2
Web presence	24	14.5	29	17.5	36	21.7
E-payment	46	27.7	51	30.7	55	33.1
E-commerce	25	15.1	30	18.1	34	20.5
**Human resources**						
Internet usage	111	66.9	124	74.7	125	75.3
Computer/Phone usage	109	65.7	120	72.3	123	74.1
Web presence	30	18.1	40	24.1	45	27.1
E-payment	40	24.1	47	28.3	49	29.5
E-commerce	22	13.3	26	15.7	29	17.5
**Marketing**						
Internet usage	144	86.7	152	91.6	154	92.8
Computer/Phone usage	138	83.1	149	89.8	151	91.0
Web presence	33	19.9	37	22.3	43	25.9
E-payment	72	43.4	79	47.6	84	50.6
E-commerce	54	32.5	58	34.9	62	37.3

*Note:* The total sample of enterprises for the services sector is 166. The frequency and percentage in the table indicate the number of enterprises out of the total sample that adopted the digital tools.

**Table 3:** Digital tool adoption in retail sector.

	Before COVID-19		During COVID-19 (MCO)		New norm	
Digital tool	Frequency	Percentage	Frequency	Percentage	Frequency	Percentage
**Finance**						
Internet usage	134	85.4	143	91.1	145	92.4
Computer/Phone usage	127	80.9	134	85.4	135	86.0
Web presence	17	10.8	24	15.3	26	16.6
E-payment	64	40.8	75	47.8	78	49.7
E-commerce	36	22.9	44	28.0	47	29.9
**Production and operations**						
Internet usage	65	41.4	70	44.6	71	45.2
Computer/Phone usage	60	38.2	65	41.4	65	41.4
Web presence	15	9.6	16	10.2	17	10.8
E-payment	23	14.6	25	15.9	28	17.8
E-commerce	15	9.6	15	9.6	18	11.5
**Human resources**						
Internet usage	87	55.4	94	59.9	97	61.8
Computer/Phone usage	80	51.0	87	55.4	88	56.1
Web presence	16	10.2	21	13.4	23	14.6
E-payment	24	15.3	25	15.9	29	18.5
E-commerce	16	10.2	17	10.8	19	12.1
**Marketing**						
Internet usage	129	82.2	134	85.4	135	86.0
Computer/Phone usage	119	75.8	123	78.3	125	79.6
Web presence	16	10.2	17	10.8	20	12.7
E-payment	47	29.9	49	31.2	54	34.4
E-commerce	43	27.4	50	31.8	54	34.4

*Note:* The total sample of enterprises for the retail sector is 157. The frequency and percentage in the table indicate the number of enterprises out of the total sample that adopted the digital tools.

**Table 4:** Digital tool adoption in manufacturing sector.

	Before COVID-19		During COVID-19 (MCO)		New norm	
Digital tool	Frequency	Percentage	Frequency	Percentage	Frequency	Percentage
**Finance**						
Internet usage	54	85.7	56	88.9	58	92.1
Computer/Phone usage	52	82.5	56	88.9	57	90.5
Web presence	12	19.0	17	27.0	23	36.5
E-payment	33	52.4	37	58.7	42	66.7
E-commerce	13	20.6	15	23.8	19	30.2
**Production and operations**						
Internet usage	46	73.0	48	76.2	51	81.0
Computer/Phone usage	44	69.8	47	74.6	50	79.4
Web presence	13	20.6	15	23.8	19	30.2
E-payment	19	30.2	21	33.3	25	39.7
E-commerce	8	12.7	9	14.3	11	17.5
**Human resources**						
Internet usage	41	65.1	45	71.4	47	74.6
Computer/Phone usage	39	61.9	43	68.3	45	71.4
Web presence	9	14.3	10	15.9	16	25.4
E-payment	12	19.0	15	23.8	18	28.6
E-commerce	7	11.1	9	14.3	10	15.9
**Marketing**						
Internet usage	57	90.5	59	93.7	62	98.4
Computer/Phone usage	55	87.3	58	92.1	61	96.8
Web presence	11	17.5	12	19.0	17	27.0
E-payment	27	42.9	29	46.0	31	49.2
E-commerce	17	27.0	18	28.6	21	33.3

*Note:* The total sample of enterprises for the manufacturing sector is 63. The frequency and percentage in the table indicate the number of enterprises out of the total sample that adopted the digital tools.

**Table 5:** Digital tool adoption in tourism sector.

	Before COVID-19		During COVID-19 (MCO)		New norm	
Digital tool	Frequency	Percentage	Frequency	Percentage	Frequency	Percentage
**Finance**						
Internet usage	37	80.4	40	87.0	41	89.1
Computer/Phone usage	32	69.6	35	76.1	36	78.3
Web presence	6	13.0	6	13.0	7	15.2
E-payment	26	56.5	30	65.2	32	69.6
E-commerce	15	32.6	19	41.3	20	43.5
**Production and operations**						
Internet usage	24	52.2	26	56.5	26	56.5
Computer/Phone usage	20	43.5	22	47.8	22	47.8
Web presence	2	4.3	3	6.5	3	6.5
E-payment	6	13.0	8	17.4	8	17.4
E-commerce	4	8.7	6	13.0	6	13.0
**Human resources**						
Internet usage	21	54.7	25	54.3	25	54.3
Computer/Phone usage	18	39.1	21	45.7	21	45.7
Web presence	4	8.7	6	13.0	7	15.2
E-payment	4	8.7	7	15.2	7	15.2
E-commerce	3	6.5	5	10.9	6	13.0
**Marketing**						
Internet usage	33	71.7	36	78.3	37	80.4
Computer/Phone usage	30	65.2	32	69.6	32	69.6
Web presence	4	8.7	5	10.9	5	10.9
E-payment	16	34.8	18	39.1	18	39.1
E-commerce	14	30.4	16	34.8	16	34.8

*Note:* The total sample of enterprises for the tourism sector is 46. The frequency and percentage in the table indicate the number of enterprises out of the total sample that adopted the digital tools.

## 2 Experimental Design, Materials and Methods

The data were collected among a sample of 432 enterprises in Malaysian industrial sectors through a self-administered online questionnaire survey hosted at SurveyMonkey.com. The survey was conducted during the periods of Conditional Movement Control Order (CMCO) and Recovery Movement Control Order (RMCO) from October 11 until December 31, 2020. It is acknowledged that the data collected over the short period of less than three months may not reveal the actual extent of digital adoption by the enterprises during the pandemic, and that data collected over a longer period during the pandemic may present the more actual rate of the digital adoption. However, it was not possible to extend the period due to an external factor that influenced the duration of data collection. The collected data, which contained nominal and ordinal values, were then coded, edited, processed, and analysed. Analysis methods used were descriptive statistics and cross-tabulation.

The sample of 432 enterprises was selected using cluster and systematic random sampling. First, a full list of registered enterprises in different industries in all Malaysian states was obtained from Malaysia's Ministry of Entrepreneur Development and Cooperatives (MEDAC). Then, enterprises under several industries were grouped together into any of the four major industrial sectors, namely services, retail, manufacturing, and tourism. Enterprises under health, education, and medicine were grouped into the services sector. Enterprises under grocery store, department store, and supermarket/hypermarket were grouped into the retail sector. Enterprises under petrochemical manufacturing, automotive, and engineering were grouped into the manufacturing sector. Those under food and beverages (F&B) and transportation were grouped into the tourism sector. Next, the number of enterprises to be sampled from each sector was calculated. The calculated number was in proportion to the total number of enterprises grouped into each sector. Finally, based on the calculated number, enterprises were randomly selected from each sector to form a sample.

The questionnaire had two sections. Section 1 contained items that asked for personal particulars of enterprises, including name, address, email, and phone number, and their type of industry, whether services, manufacturing, retail, or tourism. These items were adapted from Abdulle et al. [1], with two more sectors added as types of industry, namely retail and tourism. Section 2 contained items on digital tool adoption by the enterprises in various functions, namely finance activities, production and operations activities, human resource management activities, and marketing activities. Digital tools included as options were internet usage, computer/phone usage, web presence, e-payment, and e-commerce. These options were adapted from Tan and Chian [2]. Internet usage referred to the use of the Internet by the enterprises for their work. Computer/phone usage referred to desktops, laptops, netbooks, tablets, tablet computers, smartphones, portable or handheld computers, minicomputers, mainframes, or workstations used by enterprises for their work. Web presence referred to websites (including mobile versions) or any other web pages where enterprises have control over the content of the pages. E-payment referred to making or receiving payment through electronic means for procurement and/or sales of products and/or services. E-commerce referred to sales or purchases of goods and services over computer-mediated networks or the Internet (excluding orders received/placed by telephone, fax, or normal mail), but the payment and delivery of the goods and services can be offline. Functions included in the questionnaire were those related to finance (payment transactions, working capital management, financing management), production and operations (material handling, product design, production, quality control), human resource management (teleworking options for staff, evaluation of staff performance), and marketing (sales of goods or services, advertising). These were adapted from Temponi et al. [3]. Each item asked respondents to provide responses for three periods: before COVID-19, during COVID-19, and the new norm phase, as suggested by Papadopoulos et al. [4].

## Ethics Statement

All procedures performed in studies were in accordance with the ethical standard of Universiti Malaysia Terengganu. Informed consent was obtained from all respondents involved in the study. Participants were informed that the survey was anonymous**.**

## CRediT Author Statement

**Suriyani Muhamad:** Writing - Original draft preparation, Conceptualisation, Supervision; **Suhal Kusairi:** Methodology, Formal analysis; **Mustafa Man:** Data Retrieval; **Nur Fatma Hasni Majid:** Investigation; **Wan Zulkifli Wan Kassim**: Writing - Reviewing and Editing.

## Declaration of Competing Interest

The authors declare that they have no known competing financial interests or personal relationships which have or could be perceived to have influenced the work reported in this article.

## Data Availability

Digital Adoption by Enterprises in Malaysian Industrial Sectors During COVID-19 Pandemic: Survey Data (Original data) (Mendeley Data).

## References

[1] Abdulle A.S., Zainol Z., Ahmad Mutalib H. (2019). Impact of computerised accounting information system on small and medium enterprises in Mogadishu, Somalia: the balance scorecard perspectives. Int. J. Eng. Adv. Technol..

[2] Tan M., Chian N.W. (2019). Digital adoption among firms and impact on firm-level outcomes in Singapore. Econ. Surv Singapore First Quarter.

[3] Temponi C., Bryant M.D., Fernandez B. (2009). Integration of business function models into an aggregate enterprise system model. Eur. J. Oper. Res..

[4] Papadopoulos T., Baltas K.N., Balta M.E. (2020). The use of digital technologies by small and medium enterprises during COVID-19: Implications for theory and practice. Int. J. Inf. Manag..

